# Non-Mendelian inheritance during inbreeding of Ca_v_3.2 and Ca_v_2.3 deficient mice

**DOI:** 10.1038/s41598-020-72912-9

**Published:** 2020-10-02

**Authors:** Serdar Alpdogan, Renate Clemens, Jürgen Hescheler, Felix Neumaier, Toni Schneider

**Affiliations:** Institute for Neurophysiology, University of Cologne, Robert-Koch-Str. 39, 50931 Cologne, Germany

**Keywords:** Embryogenesis, Development

## Abstract

The mating of 77 heterozygous pairs (Ca_v_3.2[+|−] x Ca_v_3.2[+|−]) revealed a significant deviation of genotype distribution from Mendelian inheritance in weaned pups. The mating of 14 pairs (Ca_v_3.2[−|−] female x Ca_v_3.2[+|−] male) and 8 pairs (Ca_v_3.2[+|−] female x Ca_v_3.2[−|−] male) confirmed the significant reduction of deficient homozygous Ca_v_3.2[−|−] pups, leading to the conclusion that prenatal lethality may occur, when one or both alleles, encoding the Ca_v_3.2T-type Ca^2+^ channel, are missing. Also, the mating of 63 heterozygous pairs (Ca_v_2.3[+|−] x Ca_v_2.3[+|−]) revealed a significant deviation of genotype distribution from Mendelian inheritance in weaned pups, but only for heterozygous male mice, leading to the conclusion that compensation may only occur for Ca_v_2.3[−|−] male mice lacking both alleles of the R-type Ca^2+^ channel. During the mating of heterozygous parents, the number of female mice within the weaned population does not deviate from the expected Mendelian inheritance. During prenatal development, both, T- and R-type Ca^2+^ currents are higher expressed in some tissues than postnatally. It will be discussed that the function of voltage-gated Ca^2+^ channels during prenatal development must be investigated in more detail, not least to understand devastative diseases like developmental epileptic encephalopathies (DEE).

## Introduction

Calcium ions are crucial for reproduction and development^1^. Changes of cytosolic Ca^2+^ concentrations translate a diverse set of signals into specific cellular responses. More than 100 Ca^2+^ channels, pumps, exchangers, sensors and buffers contribute to the fundamental processes involved in development and propagation of living cells^2^. Voltage-gated Ca^2+^ channels (VGCCs) are a key mediator of Ca^2+^ entry from the extracellular space and enable Ca^2+^ signaling in a dual manner, electrogenically, via Ca^2+^-induced changes in membrane potential, and biochemically, through the activation of Ca^2+^ dependent enzymes and other proteins affecting cellular regulation^3^.

Ten mammalian genes are known to encode different ion conducting Ca_v_α1 subunits of these VGCCs, which have been subdivided into 7 high- and 3 low-voltage activated Ca^2+^ channels (for details,^4^). In vivo, they are assembled with additional auxiliary subunits^5^, for which the complete setup of components is only partially known. Additional structural variation arises from alternative splicing, which increases structural and functional variability^6,7^.

The ion conducting Ca_v_α1 subunits of VGCCs have been inactivated in mice to deduce their individual functions^8^. Some of the resulting mouse models are related to human diseases (for a summary see:^9^).

Several voltage-gated Ca^2+^ channels play a role in rodent models of acquired epilepsy, including the Ca_v_2.3 / R-type^10,11^ and the Ca_v_3.2/T-type channel^12,13^, both of which are highly sensitive towards divalent trace metal cations^14,15,16^. Mouse models lacking both Ca^2+^ channel types were investigated in previous studies to describe in detail their phenotypes and sensitivities towards divalent metal cations when co-injected with kainate. During the breeding of these mice the number of weaned pups did not correspond the expected ratios for a Mendelian inheritance, pointing to a possible prenatal lethality.

## Material and methods

### Material and reagents

Unless noted otherwise, all reagents were obtained from Sigma-Aldrich and used without further purification (Sigma-Aldrich Chemie GmbH, Schnelldorf, Germany)**.** Solutions were prepared with deionized, double-distilled or type I ultrapure water dispensed from an ELGA LabWater (Purelab Flex 2, United Kingdom) system respectively.

### Animals

Mice were housed at a constant temperature (20–22 °C) in makrolon type II cages, with light on from 7 a.m. to 7 p.m. (light intensity at the surface of the animal cages was between 5 and 10 lx) and ad libitum access to food and water.

The cacna1h gene encoding Ca_v_3.2 was disrupted in mice by homologous recombination^17^. These mice were inbred in C57Bl/6 background for more than 10 generations. C57Bl/6J were used as Ca_v_3.2-competent control mice. The strain abbreviation for the mouse line is C57Bl/6J-cacna1h^+|-^ for the mice lacking one Ca_v_3.2-allele (heterozygous mice) and C57Bl/6J-cacna1h^-|-^ for the mice lacking both Ca_v_3.2-alleles (homozygous mice deficient of Ca_v_3.2). Ca_v_3.2-deficient mice are available from Mutant Mouse Resource & Research Centers (MMRRC) with the strain name B6.129-Cacna1h^tm1Kcam^/Mmmh.

The cacna1e gene encoding Ca_v_2.3 was disrupted in vivo by agouti-colored Ca_v_2.3(fl| +) and deleter mice expressing Cre-recombinase constitutively^18^. Thus, exon 2 was deleted by Cre-mediated recombination. Ca_v_2.3-deficient mice were fertile, exhibited no obvious behavioral abnormalities and the born Ca_v_2.3-deficient mice had the same lifespan as control mice. Parallel breeding of parental inbred mouse lines of Ca_v_2.3-deficient and control mice ensured their identical background. The strain abbreviation for the mouse line is C57Bl/6.129SvJ-cacna1e^+|−^ for the mice lacking one Ca_v_2.3-allele (heterozygous mice) and C57Bl/6J-cacna1e^-|-^ for the mice lacking both Ca_v_2.3-alleles (homozygous mice deficient of Ca_v_2.3). Ca_v_2.3-deficient mice are available from MMRRC with the strain name B6J.129P2(Cg)-Cacna1e^tm1.1Tsch^/Mmjax.

The animal experimentation described in the text was approved by the institutional committee on animal care (Landesamt für Natur, Umwelt und Verbraucherschutz North Rhine Westfalia, Az number 84-02.04.2013.A186 and 81-02.04.2018.A176) and conducted in accordance with accepted standards of humane animal care, as described in the UFAW handbook on the care and management of laboratory animals.

### Genotyping of mice

Tail biopsies from 21 day old mice were used for the extraction of genomic DNA. Contaminating protein and RNA were enzymatically digested by protease and RNAse, respectively.

For the PCR amplification of indicative Ca_v_3.2 DNA-fragments, about 1 µg DNA was introduced and amplified with the WT-forward primer 5′- ATT CAA GGG CTT CCA CAG GGT A-3′ and the WT-reverse / KO-forward primers 5′-CAT CTC AGG GCC TCT GGA CCA C-3′ and KO-reverse primer 5′-GCT AAA GCG CAT GCT CCA GAC TG-3′^17^. The sizes of DNA fragments expected are 480 bp for the WT and 330 for the Ca_v_3.2-KOs.

For the PCR amplification of indicative Ca_v_2.3 DNA-fragments, about 1 µg DNA was introduced and amplified with the forward primer (B45Hilx1) 5′-AAA AAC AGC CGG GGA AAG CTT AT-3′ and the reverse primer (a1eb45r) 5′-CTG CCC TTT CTT CTT GCC TGA C-3′. The sizes of DNA fragments expected are 1047 bp for the WT and 86 bp for the Ca_v_2.3-KOs.

**Figure 1:**
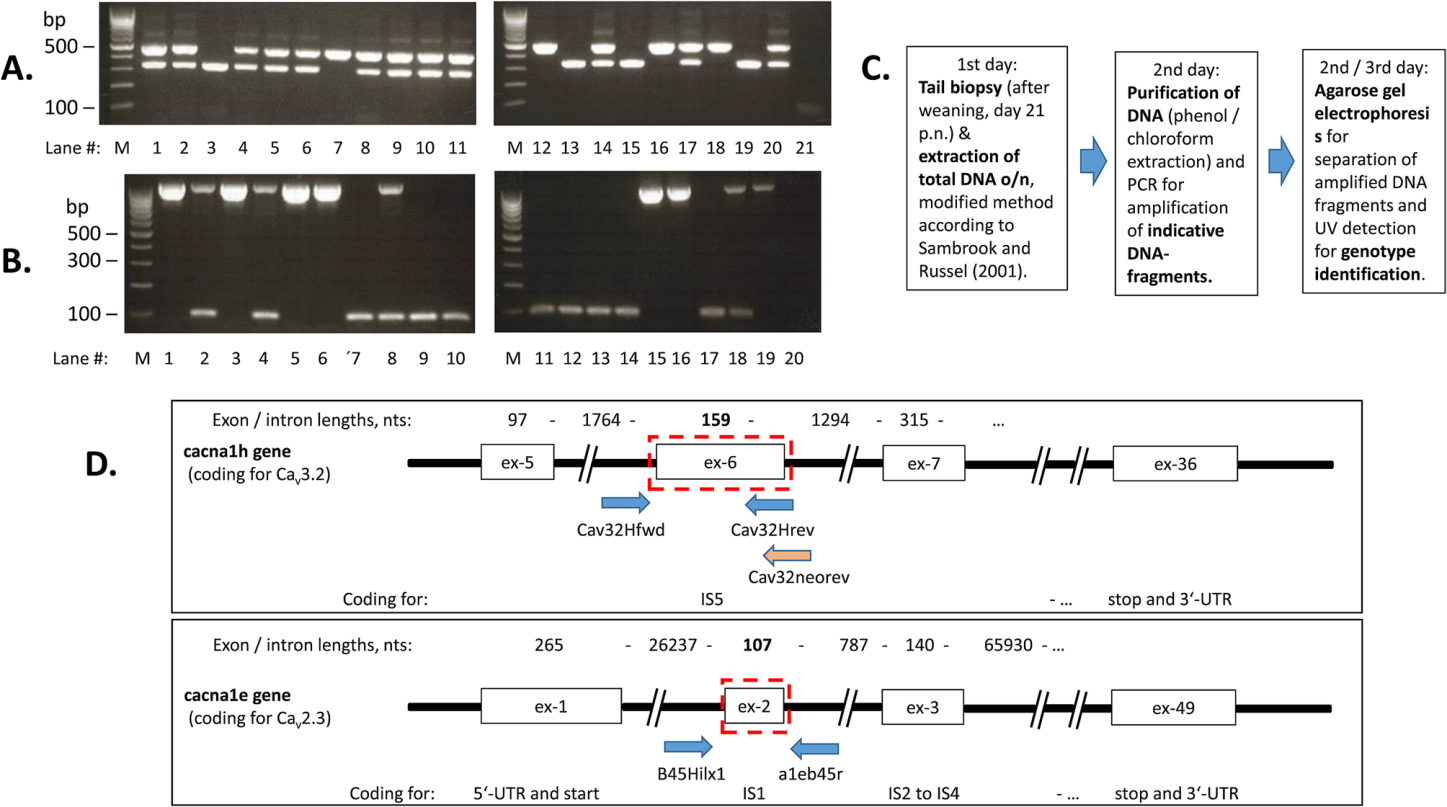
PCR-amplification of genotype specific DNA-fragments. Total DNA was isolated from tail biopsies of about 21 day old mice. M = size markers for double strand DNA, as indicated. Panel A and B are composed of two pictures, which are processed in parallel. (**A)** DNA-fragments indicative for Ca_v_3.2-competent (480 bp) and Ca_v_3.2-deficient mice (330 bp) from a typical screening experiment (lane 1 to lane 17). Double bands (480 and 330 bp) are indicative of heterozygous mice. Reference DNA from a WT control (Ca_v_3.2-competent, lane 18), from a Ca_v_3.2-KO mouse (Ca_v_3.2-deficient, lane 19), and a known heterozygous mouse (lane 20) are introduced in each screening assay. Lane 21 shows the negative control, in which no tail-DNA was added. (**B)** DNA-fragments indicative for Ca_v_2.3-competent (1056 bp) and Ca_v_2.3-deficient mice (86 bp) from a typical screening experiment (lane 1 to lane 16). Double bands (1056 and 86 bp) are indicative of heterozygous mice. Reference DNA from a WT control (Ca_v_2.3-competent, lane 17), from a Ca_v_2.3-KO mouse (Ca_v_2.3-deficient, lane 19), and a known heterozygous mouse (lane 18) are introduced in each screening assay. Lane 20 shows the negative control, in which no tail-DNA was added. (**C)** Schematic presentation the isolation of genomic DNA from tail biopsies and of genotyping by PCR. (**D)** Cartoon illustrating the gene structure, the position of primers used for genotyping and the deleted exons (dashed rectangular). *Upper panel* is showing the intron–exon structure for part of the cacna1h gene (total size 67,404 nts in humans, with 36 exons). Exon 6, encoding transmembrane segment S5 of domain I (IS5), was deleted by homologous recombination (further details: Chen CC et al., 2003), leading in the knockout allele to a novel sequence after ligation, so that only the novel reverse primer can hybridize complementarily. For the amplification of indicative cacna1h DNA-fragments from genomic DNA, the forward primer Cav32Hfwd (nts 4877–4898, Genbank ACH010580.2) and the reverse primer cav32Hrev (nts 5356–5335, GB AH010580.2) were used leading in the wild type mice to the expected fragments of 480 bp. For the Ca_v_3.2-deficient mice the same forward but another reverse primer was used (Cav32neorev) unique for the deficient mice and leading to a fragment of 330 bp. *Lower panel* is showing the intron–exon structure for part of the cacna1e gene (total size 385,835 nts, in humans with 49 exons). Exon 2, encoding transmembrane segment S1 of domain I (IS1), was deleted by homologous recombination. Exon 2 represents nts 269 to 375 (GB L29346). In total, the sequence between the HindIII- and the NsiI-site was deleted (further details: Perverzev et al., 2002). For the amplification of indicative cacna1e DNA-fragments from genomic DNA, the forward primer B45Hilx1 (nts 87151–87173, GB AC101727.8) and the reverse primer a1eb45r (nts 88198–88177, GB AC101727.8) were used leading in the wild type mice to the expected fragments of 1056 bp and in the Ca_v_23-deficient mice to a fragment of 86 bp.

PCRs for both genotypings were performed using a DNAEngine Peltier thermal cycler (BioRad, Germany) or a PTC-200 Peltier thermal cycler (MJ Research, Biozym Diagnostik, Germany) with the initial denaturation (94 °C for 10 min) followed by 34 cycles (denaturation at 94 °C for 60 s, annealing at 60 °C for 90 s, extension at 72 °C 4 min) and final extension at 72 °C for 10 min. The PCR products were separated by agarose gel electrophoresis and fluorescent bands were detected on a Herolab UVT-28 M transilluminator by UV irradiation (312 nm excitation wavelength) (Fig. 1).

### Data analysis and statistics

The assumption of normal distribution of data was tested by the Kolmogorov–Smirnov test. The Student's *t*-test was used for the comparison of two experimental groups. Data were analyzed by one-way ANOVA for multiple comparisons. Statistical analysis was performed with the GraphPad Prism software (version 8). The Mendelian genotype distributions were tested by a chi‐square test for Mendelian ratios by the use of the algorithm on the web page https://www.ihh.kvl.dk/htm/kc/popgen/genetik/applets/ki.htm. The calculated chi-square values were evaluated and converted into a probability (p-)value by using tables 4-1^19^.

### Ethical approval

All applicable international, national and institutional guidelines for the care and use of animals were followed.

## Results

During the routine breeding for Ca_v_3.2-deficient mice over a time period of 12 years, the number of genotyped knockout mice (Fig. 1) was severely under represented (the distribution for the genotypes within each group of born mice is summarized in Supplement-table S1 to S3). The consecutive systematic evaluation of wild type, heterozygous and Ca_v_3.2-deficient pups from 99 breeding pairs (Table 1) revealed a highly significant reduction of heterozygous and even more significant reduction of homozygous Ca_v_3.2-deficient mice. For comparison, the breeding history was also analyzed for the Ca_v_2.3-deficient mouse lines.

**Table 1:** Litter sizes for male and female weaned pups and deviations of genotype distribution from Mendelian inheritance.

Genotype of parents		Statistics of pups and genotype distribution					
Female(s)	Male	Mating pairs (n)	Mean litter size	Genotype deviation	Mean litter size	Genotype deviation	Sex ratioF / M
			Female pups	Chi-squared p	Male pups	Chi-squared p	
Ca_v_3.2(+|−)	Ca_v_3.2(+|−)	77	2.95 ± 0.25	< 0.01	2.97 ± 0.22	< 0.001	1.28 ± 0.17
Ca_v_3.2(−|−) Ca_v_3.2(+|−)	Ca_v_3.2(+|−) Ca_v_3.2(−|−)	14 8	1.68 ± 0.22	> 0.05	2.41 ± 0.33	<0.01	0.71 ± 0.13 (p = 0.07 *)
Ca_v_2.3(+|−)	Ca_v_2.3(−|−)	63	3.29 ± 0.21	>0.1	3.59 ± 0.21	< 0.005	1.05 ± 0.07

Mating pairs with a homozygous Ca_v_3.2(−|−) partner are summarized because of the low number of events (line in the middle of the table). * = For the summarized data in this table line, no significant difference was observed. However, in 8 matings with a homozygous male and a heterozygous female partner the number of female pups was significantly reduced (p = 0.023, Students t-test).

### Analysis of the genotypes for Ca_v_3.2/T-type mice

Ca_v_3.2 channels mediating T-type Ca^2+^ currents have been inactivated in mice by homologous recombination^17^. The deletion of exon 6 of the murine cacna1h gene was designed to delete the IS5 region in the channel protein and to impair the synthesis of a functional full-length protein. It caused a severe reduction of Ca_v_3.2 mRNA in heterozygous mice as quantified by Northern blot analysis and a complete loss of Ca_v_3.2 mRNA in Ca_v_3.2-deficient mice. In differentiated myotubes from the individual genotypes, the transcript for Ca_v_3.2 identified by RT-PCR was well detected in Ca_v_3.2-competent and completely lost in Ca_v_3.2-deficient mice. Simultaneously, the amount of transcript for Ca_v_3.1 was strongly increased in these myotubes^17^, suggesting a compensatory upregulation of these channels.

#### PCR-genotyping results

The genotyping of the litter was performed postnatally by PCR on total DNA isolated from tail biopsies (Fig. 1C,D). The amplified DNA fragments were clearly separated from each other by agarose gel electrophoresis to ensure exact genotype identification (Fig. 1A). The oligonucleotide primers were designed to detect easily and precisely DNA fragments from wild type and Ca_v_3.2 deficient mice. The intensities of DNA fragments were strong enough to identify wild type (3 mice plus 1 reference DNA), heterozygous (11 mice plus 1 reference DNA) and Ca_v_3.2-deficient candidates (3 candidates plus 1 reference DNA) (Fig. 1A). The negative control (no tail DNA included) did not contain DNA fragments of the references sizes (480 bp for wt or 330 bp for KO).

#### Distribution of individual genotypes in the mouse lines for the Ca_v_3.2 gene inactivation

During 77 breeding events from heterozygous parents, in total 83 male Ca_v_3.2( +| +) mice were born (Suppl.-Tab. 1). As null hypothesis and according to Gregor Mendel^20^, one would expect 166 heterozygous and 83 homozygous male Ca_v_3.2-deficient pups, following the law of independent assortment. But during the investigated years of breeding only 111 heterozygous and 35 Ca_v_3.2-deficient pups were born (Suppl.-Tab. 1). Similar results were achieved for the females. During the same 77 breedings, in total 72 Ca_v_3.2( +| +) female mice were born. Again, as null hypothesis with Mendelian inheritance, one would expect 144 heterozygous and 72 homozygous female Ca_v_3.2-deficient pups. However, only 116 heterozygous and 39 Ca_v_3.2-deficient pups were weaned (Suppl.-Tab. 1). Consecutively, the observed genotype distribution differed from the expected Mendelian ratio and the deviation was significant as deduced from the Chi-squared derived (CHSQ) p-values for both, males (p < 0.001) and females (p < 0.01) (Table 1).

**Figure 2:**
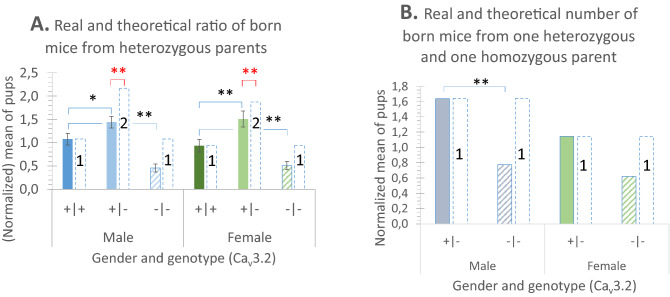
Genotype distribution profile of the offspring at the weaned stage by mating of Ca_v_3.2 parents with various allele deficiencies. The bar columns terminated by dashed lines represent the theoretically predicted numbers when a Mendelian inheritance is assumed (related to the identified number of competent ( +| +) pups). (**A)** Genotypes of the offspring from heterozygous Ca_v_3.2(+|−) parents comparing the mean number of pups per mating. (**B**) Genotypes of the offspring from the mating of one heterozygous [Ca_v_3.2(+|−)] and one homozygous [Ca_v_3.2(−|−)] partner comparing the mean number of pups per mating.

For another statistical comparison, the mean values of pups per breeding for each sex and genotype were calculated (Fig. 2A). Heterozygous mice from both sexes were still superior in number (1.3-fold for males and 1.6-fold for females), but did not reach the two-fold majority predicted from theory. When comparing the expected two-fold number with the real number of heterozygous pups, it was significantly reduced for both sexes (p = 0.0002 for males and p = 0.0023 for females) (see red stars in Fig. 2A).

When comparing the homozygous Ca_v_3.2-deficient mice from both sexes with the homozygous Ca_v_3.2-competent mice, they were significantly reduced as well (p < 0.001 for males and p = 0.009 for females) (Fig. 2A).

During 22 breeding events, one parent was heterozygote and the other homozygote. According to Gregor Mendel, half of the pups should be heterozygotes and the other half homozygote for Ca_v_3.2-deficiency. However, in both sexes, the number of Ca_v_3.2 deficient mice was reduced. While 36 heterozygous male pups were born, only 17 homozygous Cav3.2 null mice were born (for females 24 heterozygous pups and only 13 null mice). Thus, the ratio was significant reduced for the male Ca_v_3.2-deficient mice (p = 0.007 for males and p = 0.063 for females) (Fig. 2B), leading to the conclusion that the null hypothesis has to be rejected and that as an alternative hypothesis, the inactivation of the Ca_v_3.2 gene in mice may cause prenatal lethality, which does not penetrate to all but to many of the individuals.

### Analysis of the genotypes for Ca_v_2.3/R-type mice

Next, we were interested in the breeding results for Ca_v_2.3-deficient mice, which are known to exhibit a deficit in the flagellar speed of moving sperms as well as in the acrosome reaction^21,22^. The Ca_v_2.3 channels mediating R-type Ca^2+^ currents have been inactivated in mice by homologous recombination and by successive breeding with cre-deleter mice^23^. The deletion of exon 2 of the murine cacna1e gene was designed to delete the IS1 region in the channel protein and to impair the synthesis of a functional full length channel transcript. It caused the complete loss of Ca_v_2.3 channel protein as proven by Western blotting using Ca_v_2.3-selective antibodies. In heterozygous mice, the brain Ca_v_2.3 protein level was about half of the amount detected in Ca_v_2.3-competent mice^23,24^.

#### Ca_v_2.3 channels are inactivated by deleting exon 2 introducing an early stop codon

Exon 2 encoding the first transmembrane segment of the Ca_v_2.3 Ca^2+^ channel was deleted by Cre-mediated recombination^23^. After inbreeding of heterozygous parents, the genotyping of the litter was performed postnatally by PCR on total DNA isolated from tail biopsies (Fig. 1B,C). The oligonucleotide primers were designed to detect reliably DNA fragments from all genotypes. The intensities of DNA fragments were sufficiently strong to identify wild type (6 mice plus 1 reference DNA), heterozygous (3 mice plus 1 reference DNA) and Ca_v_2.3-deficient candidates (7 candidates plus 1 reference DNA) (Fig. 1B). The negative control (no tail DNA included) did not contain DNA fragments of the references sizes (1056 bp for wt or 86 bp for KO).

#### Distribution of individual genotypes in the mouse lines for the Ca_v_2.3 gene inactivation

**Figure 3:**
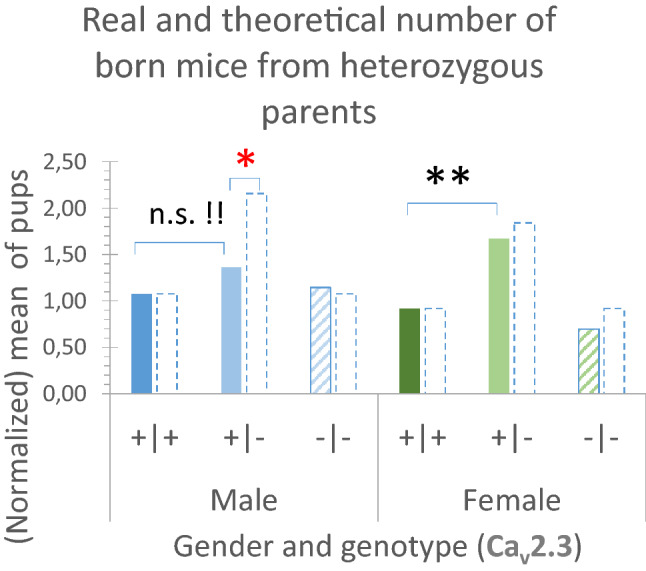
Genotype distribution profile of the offspring at the weaned stage by mating of heterozygous Ca_v_2.3(+|−) parents. The bar columns terminated by dashed lines represent the theoretically predicted numbers when a Mendelian inheritance is assumed (related to the identified number of competent ( +| +) pups). Genotypes of the offspring comparing the mean number of pups per mating. Note that non-Mendelian inheritance is restricted to the male offspring only.

During 63 breeding events from heterozygous parents, the mean litter size did not differ between male (3.6 ± 0.2) and female pups (3.3 ± 0.2) (Table 1). In total, 68 male Ca_v_3.2( +| +) mice were born (Suppl.-Tab. 3. As null hypothesis and according to Gregor Mendel^20^ one would expect 136 heterozygous and 68 homozygous male Ca_v_2.3-deficient pups. But only 86 heterozygous mice were born. Different results were achieved for the females. During the same 63 breedings, in total 58 Ca_v_2.3( +| +) female mice were born, 105 heterozygous and 44 Ca_v_2.3-deficient pups were counted (Suppl.-Tab. 3). So far, only the number of heterozygous male mice was significantly different from the expected number (CHISQ p < 0.005) (Table 1). No deviation was observed for female pups (CHISQ p > 0.1) (Table 1 and Fig. 3), leading to the conclusion that in male mice the null hypothesis has to be rejected and that as an alternative hypothesis the inactivation of one allele of Ca_v_2.3 must cause developmental problems, leading to a clear reduction of heterozygous male pups, which may only be compensated when both Ca_v_2.3 alleles are missing.

## Discussion

Our most important findings are related to deviations of genotype distributions from the expected normal Mendelian inheritance among weaned pups. For both Ca^2+^ channel types, the genotypes of the weaned offspring were significantly different from the expected Mendelian ratios.

To demonstrate that one may exclude a false genotyping, examples for the determination by PCR were included showing that indicative DNA-fragments could reliably be amplified. Further, an erroneous determination of sex can be excluded, because the sex determination was performed by an experienced coworker. If a continuous miss-determination of male heterozygous pups would have occurred, the number of heterozygote female pups must have been significantly elevated from the expected Mendelian ratio, which is not the case.

Another mistake, which could explain the “non-Mendelian ratios”, would be if unintentionally the breeding pairs would not have been all heterozygous. We can exclude it, as all parents were re-genotyped when the breeding was started. Further, we checked the data for the Ca_v_3.2-breedings and revealed in the 13 breeding lines (see Suppl.-Tab. 1) only a single breeding pair with no Ca_v_3.2 null pups. For the Ca_v_2.3-breeding data (see Suppl.-Tab. 3) we revealed in the 17 breeding lines only 2 of them, which did not have a Cav2.3 null pups.

So far, only for the ion conducting Ca_v_α1 subunit of the cardiac L-type Ca^2+^ channel a prenatal lethality is known^25^. No viable Ca_v_1.2(−|−) mice were born, but the number of heterozygous pups was normal, corresponding to the expected Mendelian ratio. The developing Ca_v_1.2-deficient pups died before day 14.5 postcoitum (p.c.) but up to day 12.5 p.c., the embryonic hearts contracted with identical frequency in wild type, heterozygous and homozygous Ca_v_1.2 deficient mice. So far, it has remained unclear, which unidentified L-type like Ca^2+^ current may enable the normal prenatal beating between day 12.5 and 14.5 p.c. in Ca_v_1.2-deficient mice^25,26^.

For the Ca_v_3.2-deficient matings, a continuous reduction in the offspring number was observed for both sexes, when one or both alleles were inactivated in the pups. It is currently unknown and would be interesting to investigate, why the lack of the Ca_v_3.2 allele causes prenatal lethality in some but not in all cases.

Ca_v_3.2 belongs to the subfamily of low-voltage activated T-type Ca^2+^ channels. They are expressed in many developing tissues and involved in regulating cell proliferation, differentiation, growth and death^27^. Both, the development of T-type channel isotypes and the development of electrophysiologically defined T-type currents reveals higher levels during embryonic states compared to the postnatal development (see Fig. 1 in^27^. There is sufficient evidence for a high expression of T-type Ca^2+^ channels in embryonic tissues at the molecular level^28^, which appears to be especially important for cardiac^29,30^ and neuronal development^31,32^.

Using information from the gnomAD data base, which quantifies the functional constraints for human genes, CACNA1H is not under significant functional constraint in the human population, though no individuals with homozygous loss of function alleles have been observed. Its o/e number with 0.38 (CI 0.28–0.5) is high, illustrating that the number of observed per expected (o/e) nucleotide variants found indicates a much higher functional constraint for CACNA1E, which is among the most constrained genes in the human genome with an o/e value of only 0.07 (CI 0.04–0.12).

While for the inherited mutations in humans, the functional constraints for the CACNA1E are much higher than for the CACNA1H gene, it does not seem to be the case for the investigated mouse models in the present study. The investigation of the role of the cacna1e gene in a neurotoxin Parkinson’s mouse model revealed that the Cav2.3 knockout even reduced activity-associated nigral somatic Ca^2+^ signals and Ca^2+^-dependent afterhyperpolarizations, leading to full protection from degeneration in vivo [2a].

On the other side, the o/e evaluation for the CACNA1E gene in the gnomAD data base fits well with the observation that de novo mutations in CACNA1E are critical. Recently, for Ca_v_2.3 in 30 children de novo gain-of-function mutations were identified, which cause developmental and epileptic encephalopathy with contractures, macrocephaly and dyskinesias^33^). These disturbances in addition cause early death in the young patients.

The ion conducting subunit Ca_v_2.3 forms the central pore of the pharmacoresistant R-type Ca^2+^ channels, which also exhibit higher expression levels during prenatal development than postnatally^34,35^. The sex specific effect of one allele loss in heterozygotes may relate to the function of Ca_v_2.3 during acrosome formation^21,22^. Sperms lacking Ca_v_2.3 show altered Ca^2+^ responses, a reduced acrosome reaction and a strong subfertility phenotype^36^. If the loss of one Ca_v_2.3 allele affects the acrosome reaction substantially, the loss of both alleles in homozygous KOs could have triggered a corresponding compensation reaction, e.g. by upregulation of another voltage-gated Ca^2+^ channel.

Probably the sex-selective deviation from the Mendelian ratio may include sex-specific hormonal effects, similar as it was reported for effects of Zn^2+^ ions on glucose homeostasis^37^.

In the literature, paradoxical inheritance with heterozygosity has been listed as one out of ten different non-Mendelian inheritance patterns^38^. Such rare cases of unusual segregation patterns are found in some specific diseases, as for example for glaucoma involving the K423E allele of *TIGR* (trabecular meshwork-inducible glucocorticoid response) gene, which is only seen in heterozygotes^39^. Obviously, the mutated proteins in homozygotes may still form functional response elements that interact with other proteins. Two additional examples are reported in the same review, which are related to a defect in the ephrin-B1 gene and to the craniofrontonasal syndrome, for which even heterozygous females are more severely affected than hemizygous mutant males^40,41^.

## Conclusion and future perspectives

Our findings show that in depth investigations are needed to understand the prenatal developmental role of voltage-gated Ca^2+^ channels. For mutations of the human Ca_v_2.3 R-type Ca^2+^ channel several gain-of-function mutations have been reported and they severely change the juvenile development during the mentioned developmental and epileptic encephalopathy^33^. A better understanding of this complex disease would help to find a better therapy for treating the children, which have a low life time expectancy.

## Supplementary information

Supplementary file1

Supplementary file2

Supplementary file3
